# The optimal range of *RET* mutations to be tested: European comments to the guidelines of the American Thyroid Association

**DOI:** 10.1186/1756-6614-6-S1-S8

**Published:** 2013-03-14

**Authors:** Laura Fugazzola, Simone De Leo, Michela Perrino

**Affiliations:** 1 Department of Clinical Sciences & Community Health , University of Milan; Endocrine Unit, Fondazione IRCCS Ca’ Granda, Via F. Sforza, 35, 20122 Milan, Italy

## Abstract

In the 9^th^ ETA-CRN Meeting (September 2009, Lisbon) some recommendations from the American Thyroid Association (ATA) guidelines for the management of medullary thyroid cancer (MTC) were discussed by an European Panel of Experts (EPE). Among the 12 ATA recommendations related to hereditary MTC and to the optimal range of *RET* mutations to be tested (recommendations 1-5 and 9-15), 7 were shared and 5 were not shared by the EPE. In the present paper, the related comments and suggestions will be reported and discussed.

## Introduction

In the 9^th^ ETA-CRN Meeting (September 2009, Lisbon) some recommendations from the American Thyroid Association (ATA) guidelines for the management of medullary thyroid cancer (MTC) [1] were discussed by an European Panel of Experts (EPE). In the present paper, the comments risen on ATA recommendations (ATA-R) related to hereditary MTC and to the optimal range of *RET* mutations to be tested will be reported and discussed (Additional file 1).

## European comments to ATA-R 1-5 and 9-15

### ATA recommendations shared by the EPE

All experts fully agreed with the need to perform RET testing in subjects at risk for autosomal dominant inheritance of MEN 2 (multiple endocrine neoplasia type 2) or FMTC (Familial Medullary Thyroid Cancer) and, in particular, shortly after birth for MEN 2B and before 5 years of age for MEN 2A and FMTC (**ATA-R #3**). Agreement was also found about the need for a pre- and post-RET testing expert genetic counseling (**ATA-R #5**) and it was stated that a list of European laboratories should probably be useful for patients. Nevertheless, though at the Orphanet EuroGentest website (http://www.orpha.net/consor/cgi-bin/index.php) more than 100 European laboratories are listed, this is probably far to be the complete list, also considering that it largely changes over times. No concerns were risen about the need to offer RET mutation analysis to all first-degree relatives of known mutation carriers (**ATA-R #9**). The topic of **ATA-R #12** relates to the indication for the sequencing of the entire coding region of RET in patients with MEN 2 and negative for mutations in exons 8, 10, 11, 13-16. It should be underlined that the remaining 13 exons (1-7, 9, 12, 17-21) have never been found to be mutated [2]. Nevertheless, though this approach was considered poorly cost effective, most experts and the 46% of the participants agreed with the ATA indications, since rare mutations comprise up to 30% of all hereditary cases, based on the literature data [3-5]. An additional recommendation would be to start the analysis of the additional regions of the gene from exon 5, which has been reported to be mutated (G321R) [6]. Strictly related to this topic, the possibility to analyze, in this category of patients, pheocrocytoma (PHAEO)- related genes was also discussed. The background was that 40-45% of apparently sporadic PHAEO or paragangliomas are hereditary and germline mutations are found in VHL (von Hippel-Lindau tumor suppressor, E3 ubiquitin protein ligase), SDHB/C/D (succinate dehydrogenase complex, subunit B, subunit C, subunit D) and RET (ret proto-oncogene) [7]. Nevertheless, the prevalence of mutated cases is extremely variable among different Countries, and in particular very low for the RET gene. This must be considered when the decision is taken to analyze PHAEO-related genes in RET negative cases.

In the clinical setting of MEN 2B, since virtually all patients harbor mutations in exons 15 and 16 (A883F and M918T, respectively), the panel agreed with ATA indication to analyze both exons and to test the entire *RET* coding region if those 2 exons are negative for mutations **(ATA-R #13 and #14)**. Finally, total agreement was also expressed regarding the **ATA-R #15** which indicates to treat MEN 2B patients harboring codon 804 mutation associated to another mutation similarly to those with the more typical MEN 2B *RET* causing mutations. Indeed, data from the literature report that the phenotype associated to V804M mutation alone is mostly FMTC [8,9], though also MEN 2A cases have been described [9-11]. However, when this mutation is associated to another on the same allele (Y806C, S904C, E805K, V778I) the phenotype resembles that of MEN 2B [12-16].

### ATA recommendations not shared by the EPE

The **ATA-R #1**, related to the indication to offer the germline *RET* testing to all patients with a personal medical history of primary C cell hyperplasia (CCH), MTC, or MEN2, was extensively discussed. In particular, while no concerns were risen about the need to perform *RET* analysis in a patient with MEN, it was observed that *RET* mutations are extremely rare in patients with CCH (0.03%), as reported by Scheuba et al. in 2009 [17]. Thus, the genetic analysis was not considered cost-effective for CCH cases by about half of the EPE, though the indication to recommend it was shared by other experts and by the majority of the participants. Similarly, there were some pros and cons also regarding apparently sporadic MTC. On one hand, the presentation of sporadic and familial-non MEN2A /2B- forms is almost identical and they cannot be differentiated on a clinical basis. Moreover, it has been reported that 6.5-10% of apparently sporadic cases are indeed hereditary forms [6,18]. On the other hand, it was outlined that the extensive and non-selective use of *RET* testing could lead to a stop in the reimbursement of the genetic screening (at least of the less involved exons) by the National Health Care Systems in patients with MTC, citing a lack of evidence to suggest hereditary disease [19]. The final recommendation was thus to modulate *RET* testing in apparently sporadic MTC according to clinical data and the local General Health System.

The **ATA-R #11** is related to the identification of the optimal range of *RET* mutations to be tested. Most of the experts and about half of the participants suggested to extend the analysis to exon 8, at least in all regions where mutations in that exon have been described, always in non MEN 2A/2B hereditary MTCs (Brazil, Greece, France, Italy) [20-23].

**ATA-R #10** (grade A) suggests to perform the testing of exon 10 in individuals with Hirschprung disease (HSCR), in order to detect families with activating mutations 618 o 620 in whom HSCR and MEN 2A are associated. Nevertheless, it should be highlighted that this association is frequent for MEN 2A (25-40% of families), but it is rare (2%) [24] in a very common disease such as HSCR, which has been related to mutations in at least 10 different genes. *RET* testing was thus considered to be not cost-effective and most experts indicated to downgrade this recommendation, and proposed to test HSCR patients for basal Ct.

Another important topic was related to the lichen planus amyloidosis, in the presence of which ATA guidelines recommend (**#4**, grade C) to perform the *RET* genetic testing for 634 mutation. The EPE and the large majority of participants proposed to upgrade this recommendation based on the data of the literature showing that this early cutaneous manifestation is more frequent than previously suspected, being present in up to 36% of MEN 2A cases [25]. In addition, lichen planus has been associated not only to codon 634, but also to codon 804 mutations [26]. Thus, the presence of lichen planus amyloidosis or pruritis in the central upper back should prompt Ct measurement in adults and genetic testing in children, possibly after 4 mm punch biopsy and staining with thioflavin T in order to identify amyloid deposits.

**ATA-R #2** (grade B) is related to the indication to perform *RET* testing in cases of intestinal ganglioneuromatosis. The background refers to data reporting that in MEN 2B, de novo mutations are extremely more frequent (50-90%) than for the other familial MTCs (2-9%) [27]. For this reason, the early diagnosis of MEN 2B in several cases cannot be based on familial *RET* screening. Moreover, extrathyroidal MEN 2B symptoms develop age dependently, and, though the majority of the “classic” symptoms is not expressed before 4-6 years of age, ganglioneuromatosis is always present and it is one of the earliest signs [28]. In keeping with the general agreement that in MEN 2B the clinical diagnosis of the syndrome is crucial, EPE was concordant in the indication to perform the *RET* screening in cases presenting with one or more signs related to the syndromes, such as ganglioneuromatosis, but also bumpy lips with mucosal neuroma, and corneal nerves thickening (with a prevalence of 69% in MEN 2B and 29% in MEN 2A) [29,30]. Another crucial sign, not reported in the ATA guidelines, is the tearless crying which can be recognized in more than 85% of the MEN 2A/B infants and which seems to be one of the most promising signs [27], though more data are likely needed to include this manifestation as indication for *RET* testing in infants without any other MEN 2 symptoms and with negative family history.

Finally, the EPE discussed the opportunity to include some recommendation about *RET* polymorphism, not reported in the ATA guidelines. Conflicting results have been published in recent years about the possible role of *RET* polymorphisms as genetic modifiers either in sporadic or hereditary MTC. In particular, and only considering familial MTCs, an association between G691S and S904S with an earlier age at onset in MEN2A [31] was observed, in patients with the V804M mutation, the association between L769L and earlier age of onset was reported [32], whereas in patients with G533C mutation, the variant IVS1-126G > T was found to be associated with age at diagnosis and IVS8 +82A > G; 85-86 insC with node metastases at diagnosis [33]. More recently, the RET S836S variant has been found to be associated with early onset and increased risk for metastatic disease [34]. Nevertheless, the data available to date have not been significantly replicated and discordant data have been obtained in different series [35]. Thus, the EPE and most of the participants supported the idea to draw a recommendation highlighting the lack of clinical significance of polymorphic variants.

## List of abbreviations used

CCH: C cell hyperplasia; FMTC: Familial Medullary Thyroid Cancer; HSCR: Hirschprung disease; MEN 2: multiple endocrine neoplasia type 2; MTC: Medullary Thyroid Cancer; PHAEO: pheocrocytoma

## Competing interests

No competing interests exist for me and my co-authors.

## Supplementary Material

Additional file 1 Table 1. American Thyroid Association recommendations (ATA R) and the corresponding recommendation resulting from the European Panel of Experts (EPE R rev). Click here for file

## Declarations

This article has been published as part of *Thyroid Research* Volume 6 Supplement 1, 2013: European comments on Medullary Thyroid Cancer Management Guidelines of the American Thyroid Association. The full contents of the supplement are available online at http://www.thyroidresearchjournal.com/supplements/6/S1. Publication of this supplement has been supported by the European Thyroid Association-Cancer Research Network.
