# Supplementary material for: The optimal range of RET mutations to be tested: European comments to the guidelines of the American Thyroid Association
Source: Thyroid Res. 2013 Mar 14;6(Suppl 1):S8. doi: 10.1186/1756-6614-6-S1-S8 (PMC3599734; doi:10.1186/1756-6614-6-S1-S8)
Supplement: Additional file 1 — Table 1. American Thyroid Association recommendations (ATA R) and the corresponding recommendation resulting from the European Panel of Experts (EPE R rev). [file 1756-6614-6-S1-S8-S1.docx]

**Table 1:** American Thyroid Association recommendations (**ATA R**) and the corresponding recommendation resulting from the European Panel of Experts (**EPE R rev**)

| **ATA R1** All patients with personal medical history of primary C cell hyperplasia, MTC, or MEN2 should be offered germline RET testing (grade A) |
| --- |
| **EPE R1 rev** The genetic analysis is recommended in all cases with personal medical history of MEN (grade A). In patients with a personal history of C cell hyperplasia or MTC, the RET testing should be modulated according to clinical data and the local General Health System, considering that genetic alterations will be found in <0.5 and 4-10%, respectively (grade C) |
| **ATA R2** The differential diagnosis in patients with intestinal ganglioneuromatosis should include MEN 2B, which together with their history and physical examinations, family history, and ganglioneuromatosis histology may prompt germline RET testing (grade B) |
| **EPE R2 rev** Ganglioneuromatosis, mucosal neuromas, corneal nerve thickening and probably tearless crying should be considered early signs of MEN2 and prompt germline RET testing (grade A) |
| **ATA R3** All people with a family history consistent with MEN 2 or FMTC, and at risk of autosomal dominant inheritance of the syndrome, should be offered RET testing. For MEN 2B this should be done shortly after birth. For MEN 2A and FMTC this should be done before 5 years of age (grade A) |
| **EPE R3 rev** Total agreement (grade A) |
| **ATA R4** Lichen planus amyloidosis or pruritis in the central upper back may indicate the presence of a 634 codon mutation and should prompt genetic testing (grade C) |
| **EPE R4 rev** the presence of lichen planus amyloidosis or pruritis in the central upper back should prompt Ct measurement in adults and genetic testing in children, possibly after 4 mm punch biopsy and staining with thioflavin T in order to identify amyloid deposits (grade A) |
| **ATA R5** Pre- and post-test genetics counseling by a genetics counselor, or other qualified professional, should be offered to all patients undergoing RET testing (grade C) |
| **EPE R5 rev** Total agreement (grade B) |
| **ATA R9** Once a germline RET mutation has been identified in a family, RET mutation analysis should be offered to all first-degree relatives of known mutation carriers which should be done before the age of recommended prophylactic thyroidectomy whenever possible (grade A) |
| **EPE R9 rev** Total agreement (grade A) |
| **ATA R10** Testing of exon 10 for activating RET mutations should be considered in individuals with Hirschprung disease (grade A) |
| **EPE R10 rev** In patients with HCSR disease, measurement of serum calcitonin should be performed. If elevated, RET exon 10 should be tested (grade C) |
| **ATA R11** analysis of the MEN 2-specific exons of RET is the recommended method of initial testing in either a single or multi-tiered approach (grade A) |
| **EPE R11 rev** Systematic screening for RET mutations in exon 10, 11, 13-16 represents the current gold standard and should be completed by exon 8 analysis (grade A) |
| **ATA R12** Sequencing the entire coding region of RET to identify MTC causative mutations should be done when the analysis using the recommended method is negative in the clinical setting of MEN 2 or when there is a discrepancy between genotype and phenotype (grade B) |
| **EPE R12 rev** Agreement. The analysis should start from exon 5. In selected cases PHAEO-related genes could be analyzed (grade B) |
| **ATA R13** Testing of patients with MEN2B should include analyses to detect the M918T (exon 16) and A883F mutations (exon 15) present in virtually all of the patients (grade A) |
| **EPE R13 rev** Total agreement (grade A) |
| **ATA R14** In the clinical setting of MEN 2B and negative testing for M918T and A883F mutations, sequencing the entire coding region of RET should be performed (grade B) |
| **EPE R14 rev** Total agreement (grade A) |
| **ATA R15** Until the phenotype of MEN 2B associated with codon 804 mutations in conjunction with a second variant in RET is clarified, these patients and mutation carriers should be treated similarly to those with the more typical MEN 2B RET-causing mutations (grade C) |
| **EPE R15 rev** Total agreement (grade C) |
| **EPE R ins (topic: RET polymorphisms)** To date, no definite clinical significance can be given to the presence/absence of RET polymorphic variants, but data are still insufficient to draw a definite recommendation |
